# Supplementary material for: Heat adaptation of phage T7 under an extended genetic code
Source: Virus Evol. 2021 Dec 1;7(2):veab100. doi: 10.1093/ve/veab100 (PMC8923235; doi:10.1093/ve/veab100)
Supplement: veab100_Supp [file veab100_supp.zip › Sequencing_Analysis.zip!/Sequencing_Analysis/S2/evidence/JC_SIDE_1_55.html]

BRESEQ :: Evidence


| New junction evidence | | | | | | | | | | | |
| --- | --- | --- | --- | --- | --- | --- | --- | --- | --- | --- | --- |
|  | | seq id | position | reads (cov) | reads (cov) | score | skew | freq | annotation | gene | product |
| \* | ? | NC\_001604.1 | = 8335 | 5558 (1.050) | 25 (0.000) | 22/584 | 44.4 | 0.45% | intergenic (–/–) | *–/–* | –/– |
|  | ? | NC\_001604.1 | 8939 = | 5643 (1.100) |  |  |  |  | intergenic (–/–) | *–/–* | –/– |
| Rejected: Coverage evenness skew score above cutoff. | | | | | | | | | | | |
| Rejected: Frequency below/above cutoff threshold. | | | | | | | | | | | |

Only 100 of 5580 total aligned reads displayed.  

`AGCGTCCACATCATTATCACCCGTGGTGACTTTGAGAAAGCAATAGACAAGAAACGTCCGGTTCTTAGTGTGGCAGTGACCCGCTTCCCGTTCGTCCGTCTGTTACTCAAACGAATCAAGGAGGTGTTCTGATGGGACTGTTAGATGGTGAAGCCTGGGAAAAAGAAAACCCGCCAGTACAAGCAACTGGGTGTATAGCTTGCTTAGAGAAAGATGACCGTTATCCACACACCTGTAACAAAGGAGCTAACGATATGACCGAACGTGAACAAGAGATGATCATTAAGTTGATAGACAATAATGAAGGTCGCCCAGATGATTTGAATGGCTGCGGTATTCTCTGCTCCAATGTCCCTTGCCACCTCTGCCCCGCAAATAACGATCAAAAGATAACCTTAGGTGAAATCCGAGCGATGGACCCACGTAAACCACATCTGAATAAACCTGAGGTAACTCCTACAGATGACCAGCCTTCCGCTGAGACAATCGAAGGTGTCACTAAGCCTTCCCACTACATGCTGTTTGACGACATTGAGGCTATCGAAGTGATTGCTCGTTCAATGACCGTTGAGCAGTTCAAGGGATACTGCTTCGGTAACAT  >  NC_001604.1/8035‑8635`  

                                                                                                                                                                                                                                                                                                            `|`                                                                                                                                                                                                                                                                                                              
   `gtcggagatgtgtataagagaaaggACTTTGAGAAAGCAATAGACAAGAAACGTCCGGTTCTGAGTGTGGCAGTGACCCGCTTCCCGTTCGTCCGTCTGTTACTCAAACGAATCAAGGAGGTGTTCTGATGGGACTGTTAGATGGTGAAGCCTGGGAAAAAGAAAACCCGCCAGTACAAGCAACTGGGTGTATAGCTTGCTTAGAGAAAGATGACCGTTATCCACACACCTGTAACAAAGGAGCTAACGATATGACCGAACGTGAACAAGAGATGATCATTAAGTTGATAGACAATAATGa                                                                                                                                                                                                                                                                                                           <  1:599032/277‑1 (MQ=255)`  
   `gTCCACATCATTATCACCCGTGGTGACTTTGAGAAAGCAATAGACAAGAAACGTCCGGTTCTTAGTGTGGCAGTGACCCGCTTCCCGTTCGTCCGTCTGTTACTCAAACGAATCAAGGAGGTGTTCTGATGGGACTGTTAGATGGTGAAGCCTGGGAAAAAGAAAACCCGCCAGTACAAGCAACTGGGTGTATAGCTTGCTTAGAGAAAGATGACCGTTATCCACACACCTGTAACAAAGGAGCTAACGATATGACCGAACGTGAACAAGAGATGATCATTAAGTTGATAGACAATAATGa                                                                                                                                                                                                                                                                                                           >  1:36733/1‑301 (MQ=255)`  
        `catcatTATCACCCGTGGTGACTTTGAGAAAGCAATAGACAAGAAACGTCCGGTTCTTAGTGTGGCAGTGACCCGCTTCCCGTTCGTCCGTCTGTTACTCAAACGAATCAAGGAGGTGTTCTGATGGGACTGTTAGATGGTGAAGCCTGGGAAAAAGAAAACCCGCCAGTACAAGCAACTGGGTGTATAGCTTGCTTAGAGAAAGATGACCGTTATCCACACACCTGTAACAAAGGAGCTAACGATATGACCGAACGTGAACAAGAGATGATCATTAAGTTGATAGACAATAATGAAGGTc                                                                                                                                                                                                                                                                                                      >  1:110007/1‑301 (MQ=255)`  
          `tcatTATCACCCGTGGTGATTTTGAGAAAGCAATAGACAAGAAACGTCCGGTTCTTAGTGTTGCATTGACCCGCTTCCCGTTCGTCCGTCTGTTACTCAAACGAATCAAGGAGGTGTTCTGATGGGTCTGTTAGATGGTGAAGCCTGGGAAAAAGATAACCCGCCAGTACAAGCAACTGGTTTTATAGCTTGCTTAGATAAAGATTACCATTATCCACACAACTTTAACAAAGGAGCTCACTATATGACCTAACATGAACAAGATATGATCATTAAGTTTATATCCAATACTGATGTTCGc                                                                                                                                                                                                                                                                                                    >  1:527600/1‑301 (MQ=255)`  
          `tcatTATCACCCGTGGTGACTTTGAGAAAGCAATAGACAAGAAACGTCCGGTTCTTAGTGTGGCAGTGACCCGCTTCCCGTTCGTCCGTCTGTTACTCAAACGAATCAAGGAGGTGTTCTGATGGGACTGTTAGATGGTGAAGCCTGGGAAAAAGAAAACCCGCCAGTACAAGCAACTGGGTGTATAGCTTGCTTAGAGAAAGATGACCGTTATCCACACACCTGTAACAAAGGAGCTAACGATATGACCGAACGTGAACAAGAGATGATCATTAAGTTGATAGACAATAATGAAGGTCGc                                                                                                                                                                                                                                                                                                    >  1:710438/1‑301 (MQ=255)`  
            `atTATCACCCGTGGTGACTTTGAGAAAGCAATAGACAAGAAACGTCCGGTTCTTAGTGTGGCAGTGACCCGCTTCCCGTTCGTCCGTCTGTTACTCAAACGAATCAAGGAGGTGTTCTGATGGGACTGTTAGATGGTAAAGCCTGGGAAAAAGAAAACCCGCCAGTACAAGCAACTGGGTGTATAGCTTGCTTAGAGAAAGATGACCGTTATCCACACACCTGTAACAAAGGAGCTAACGATATGACCGAACGTGAACAAGAGATGATCATTAAGTTGATAGACaataagctgtctcttat                                                                                                                                                                                                                                                                                                  >  1:345333/1‑289 (MQ=255)`  
                     `cGTGGTGACTTTGAGAAAGCAATATACAAGAAACGTCCGGTTCTTAGTGTGGCAGTGACCCGCTTCCCGTTCGTCCGTCTGTTACTCAAACGAATCAAGGCGGTGTTATGATGGGACTGTTCGATGGTGAAGACTGGGTCAAAGAAAACCCGCCAGTACCAGCAACTGGGTGTATCGCTTGCTTAGAGAAAGATGACCGTTATCCACACCCCTTTAACATAGGAGCTACCGATATGACCGAACGTGCACCAGAGATGATCATTAATTTGATAGACAATACTGactgtatcttctacccttt                                                                                                                                                                                                                                                                                         >  1:480611/1‑283 (MQ=255)`  
                         `gtgACTTTGAGAAAGCAATAGACAAGAAACGTCCGTTTCTTAGTGTGGCAGTGACCCGCTTCCCGTTCGTCCGTCGGTTACTCAAACGAATCAAGGAGGTGTTCTGATGGGACTGTTAGATGGTGAAGCCTGGGAAAAAGAAAACCCGCCAGTACAAGCAACTGGGTGTATAGCTTGCTTAGAGAAAGATGACCGTTATCCACACACCTGTAACAAAGGAGCTAACGATATGACCGAACGTGAACAAGAGATGATCATTAAGTTGATAGACAATAATGAAGGTCGCCCAGATGATTTGAAt                                                                                                                                                                                                                                                                                     <  1:352486/301‑1 (MQ=255)`  
                         `gtgACTTTGAGAAAGCAATAGACAAGAAACGTCCGGTTCTTAGTGTAGCAGTGACCCGCTTCCCGTTCGTCCGTCTGTTACTCAAACGAATCAAGGAGGTGTTCTGATGGGACTGTTAGATGGTGAAGCCTGGGAAAAAGAAAACTCGCCAGTACAAGCAACTGGGTGTATAGCTTGCTTAGAGAAAGATGACCGTTATCCACACACCTGTAACAAAGGAGCTAACGATATGACCGAACGTGAACAAGAGATGATCATTAAGTTGATAGACAATAATGAAGGTctgtctcttatacacatc                                                                                                                                                                                                                                                                                     >  1:780276/1‑284 (MQ=255)`  
                                 `gagaAAGCAATAGACAAGAAACGTCCGGTTCTTAGTGTGGCAGTGACCCGCTTCCCGTTCGTCCGTCTGTTACTCAAACGAATCAAGGAGGTGTTCTGATGGGACTGTTAGATGGTGAAGCCTGGGAAAAAGAAAACCCGCCAGTACAAGCAACTGGGTGTATAGCTTGCTTAGAGAAAGATGACCGTTATCCACACACCTGTAACAAAGGAGCTAACGATATTACCGAACGTGAACAAGAGATGATCATTAAGTTGATAGACAATAATGAAGGTCGCCCAGATGATTTGAATGGCTGCgg                                                                                                                                                                                                                                                                             <  1:673387/301‑1 (MQ=255)`  
                                   `aaaaGGAATTGACAAGAACCGTCCGGTTCTTAGTGTGGCAGTGACCCGCTTCCCCTTCGTCCGTCTTTTACCCCAACGAATCAAGGAGGTGTTCTGATGGGACTGTTAAATGGTGAAGCCTGGGAAAAAGAAAACCCTCCAGTACAAGCCACTGGGTGTATAGCTTGCTTAGAGAAAGATGACCGTTATCCACACACCTGTAACAAAGGAGCTAACGATATGACCGAACGTGAACAAGAGATGATCATTAAGTTGATAGACAATAATGAAGGTCGCCCAGATGATTTGAATGGCTGCGGTa                                                                                                                                                                                                                                                                           <  1:716810/300‑1 (MQ=255)`  
                                      `agaaaTAGAAAAGAAACGGCCGGTTTTTCGTGGGGCAGTGACACGCTTCCCGTTCGTCCGTCGATTAATCAAACGAATCAAGGAGGTTTTCTGATGGGACGGTTAGATGGTGAAGCCTGGGAAAAAGAAAACCCGCCAGTACAAGCAACTGGGTGTATAGCTTGCTTAGAGAAAGATGACCGTTATCCACACACCTGTAACAAAGGAGCTAACGATATGACCGAACGTGAACAAGAGATGATCATTAAGGTGATAGACAATAATGAAGGTCGCCCAGATGATTTGAATGGCTGCGGTATtc                                                                                                                                                                                                                                                                        <  1:450782/298‑1 (MQ=255)`  
                                        `aaaTAGACAAGAAACGTCCGGTTCGTAGTGTGGCAGTGACCCGCTTCCCGTTCGTCCGTCTGTTACTCAAACGAATCAAGGAGGTGTTCTGATGGGACTGTTAGATGGTGAAGCCTGGCAAAAAGAAAACCCGCCAGTACAAGCAACTGGGTGTATAGCTTGCTTAGAGAAAGATGACCGTTATCCACACACCTGTAACAAAGGAGCTAACGATATGACCGAACGTGAACAAGAGATGATCATTAAGTTGATAGACAATAATGAAGGTCGCCCAGATGATTTGAATGGCTGCGGTATtctc                                                                                                                                                                                                                                                                      <  1:395566/300‑1 (MQ=255)`  
                                         `aaTAGACAAGAACCGTCCGGTTCTTAGTGTGGCAGTGACCCGCTTCCCGTTCGTCCGTCTGTTACTCAAACGAAGCAAGGAGGTGTTCTGATGGGACTGTTAGATGGTGAAGCCTGGGAAAAAGAAAACCCGCCAGTACAAGCAACTGGGTGTATAGCTTGCTTAGAGAAAGATGACCGTTATCCACACACCTGTAACAAAGGAGCTAACGATATGACCGAACGTGAACAAGAGATGATCATTAAGTTGATAGACAATAATGAAGGTCGCCCAGATGATTTGAATGGCTGCGGTATTctct                                                                                                                                                                                                                                                                     <  1:702138/301‑1 (MQ=255)`  
                                             `gACAAGAAACGTCCGGTTCTTAGTGTGGCAGTGACCCGCTTCCCGTTCGTCCGTCTGTTACTCAAACGAATCAAGGAGGTGTTCTGATGGGACTGTTAGATGGTGAAGCCTGGGAAAAAGAAAACCCGCCAGTACAAGCAACTGGGTGTATAGCTTGCTTAGAGAAAGATGACCGTTATCCACACACCTGTAACAAAGGAGCTAACGATATGACCGAACGTGAACAAGAGATGATCATTAAGTTGATAGACAATAATGAAGGTCGCCCAGATGATTTGAATGGCTGCGGTATTCTCTGCTc                                                                                                                                                                                                                                                                 >  1:651847/1‑301 (MQ=255)`  
                                             `aaCAAGAAACGTCCGGTTCTTAGTGTGGCAGTGACCCGCTTCCCGTTCGTCCGTCTGTTACTCAAACGAATCAAGGAGGTGTTCTGATGGGACTGTTAGATGGTGAAGCCTGGGAAAAAGAAAACCCGCCAGTACAAGCAACTGGGTGTATAGCTTGCTTAGAGAAAGATGACCGTTATCCACACACCTGTAACAAAGGAGCTAACGATATGACCGAACGTGAACAAGAGATGATCATTAAGTTGATAGACAATAATGAAGGTCGCCCAGATGATTTGAATGGCTGCGGTATTCTCTGCTc                                                                                                                                                                                                                                                                 <  1:489406/300‑1 (MQ=255)`  
                                              `aCAAGAACCGTCCGGTTCTTAGTGTGGCAGTGACCCGCTTCCCGTTCGTCCGTCTGTTACTCAAACGAATCAAGGAGGTGTTCTGATGGGACTGTTAGATGGTGAAGCCTGGGAAAAAGAAAACCCGCCAGTACAAGCAACTGGGTGTATAGCTTGCTTAGAGAAAGATGACCGTTATCCACACACCTGTAACAAAGGAGCTAACGATATGACCGAACGTGAACAAGAGATGATCATTAAGTTGATAGACAATAATGAAGGTCGCCCAGATGATTTGAATGGCTGCGGTATTCTCTGCTcc                                                                                                                                                                                                                                                                <  1:77089/301‑1 (MQ=255)`  
                                                `acgagaCGGCCGGTTCTTAGTGTGGCAGTGACCCGCTTCCCGTTCGTCCGTCTGTTACTCAACCGAATCAAGGAGGTGTTCTGATGGGACTGTTAGATGGTGAAGCCTGGGAAAAAGAAAACCCGCCAGTACAAGCAACTGGGTGTATAGCTTGCTTAGAGAAAGATGACCGTTATCCACACACCTGTAACAAAGGAGCTAACGATATGACCGAACGTGAACAAGAGATGATCATTAAGTTGATAGACAATAATGAAGGTCGCCCAGATGATTTGAATGGCTGCGGTATTCTCTGCTCCaa                                                                                                                                                                                                                                                              <  1:165068/296‑1 (MQ=255)`  
                                                   `aaaCGTCCGGTTCTTAGTGTGGCAGTGACCCGCTTCCTGTTCGTCCGTCTGTTACTCAAACGATTCAAGGAGGTGTTCTGATGGGACTGGTAGATGGTGAAGCCTGGGAAAAAGAAAACCCGCCAGTACAAGCAACTGGGTGTATAGCTTGCTTAGAGAAAGATGACCGTTATCCACACACCTGTAACAAAGGAGCTAACGATATGACCGAACGTGAACAAGAGATGATCATTAAGTTGATAGACAATAATGAAGGTCGCCCAGATGATTTGAATGGCTGCGGTATTCTCTGCTCCAATGt                                                                                                                                                                                                                                                           <  1:187217/301‑1 (MQ=255)`  
                                                       `gacaggTTCTTAGTTTGGCAGTGACCCGCTTCCCGTTCGTCCGTCTGTTACTCAAACGAATCAAGGAGGTGTTCTGATGGGACTGTTAGATGGTGAAGCCTGGGAAAAAGAAAACCCGCCAGTACAAGCAACTGGGTGTATAGCTTGCTTAGAGAAAGATGACTGTTATCCACACACCTGTAACAAAGGAGCTAACGATATGACCGAACGTGAACAAGAGATGATCATTAAGTTGATAGACAATAATGAAGGTCGCCCAGATGATTTGAATGGCTGCGGTATTCTCTGCTCCAATGTCCCt                                                                                                                                                                                                                                                       <  1:338425/297‑1 (MQ=255)`  
                                                           `ggTTCTTAGTGTGGCAGTGACCCGCTTCCCGTTCGTCCGTCTGTTACTCAAACGAATCAAGGAGGTGTTCTGATGGGACTGTTAGATGGTGAAGCCTGGGAAAAAGAAAACCCGCCAGTACAAGCAACTGGGTGTATAGCTTGCTTAGAGAAAGATGACCGTTATCCACACACCTGTAACAAAGGAGCTAACGATATGACCGAACGTGAACAAGAGATGATCATTAAGTTGATAGACAATAATGAAGGTCGCTCAGATGATTTGAATGGCTGCGGTATTCTCTGCTCCAATGTCCCTTTcc                                                                                                                                                                                                                                                   >  1:725231/1‑301 (MQ=255)`  
                                                            `gTTCTTAGTGTGGCAGTGACCCGCTTCCCGTTCGTCCGTCTGTTACTCAAACGAATCAAGGAGGTGTTCTGATGGGACTGTTAGATGGTGAAGCCTGGGAAAAAGAAAACCCGCCAGTACAAGCAACTTGGTGTATAGCTTGCTTAGAGAAAGATGACCGTTATCCACACACCTGTAACAAAGAAGCTAACAATATGACCGAACGTGAACAAGAGATGATCATTAAGTTGATAGACAATAATGAAGGTCGCACAGATGAGTTGAATGGCTGCGGTATTCTCAGCCCCCATGTCACTTGCCa                                                                                                                                                                                                                                                  >  1:764275/1‑301 (MQ=255)`  
                                                            `gTTCTTAGTGTGGCAGTGACCCGCTTCCCGTTCGTCCGTCTGTTACTCAAACGAATCAAGGAGGTGTTCTGATGGGACTGTTAGATGGTGAAGCCTGGGAAAAAGAAAACCCGCCAGTACAAGCAACTGGGTGTATAGCTTGCTTAGAGAAAGATGACCGTTATCCACACACCTGTAACAAAGGAGCTAACGATATGACCGAACGTGAACAAGAGATGATCATTAAGTTGATAGAAAATAATGAAGGTCGCCCAGATGATTTGAATGGCTGCGGTATTCTCTGCTCCAATTTCCCTTGcaa                                                                                                                                                                                                                                                  >  1:85156/1‑299 (MQ=255)`  
                                                                   `gtgtGGCAGTGACCCGCCTCCCGTTCGTCCGTCTGTTACTCAAACGAATCAAGGAGGTGTTCTGATGGGACTGTTAGATGGTGAAGCCTGGGAAAAAGAAAACCCGCCAGTACAAGCAACTGGGTGTATAGCTTGCTTAGAGAAAGATGACCGTTATCCACACACCTGTAACAAAGGAGCTAACGATATGACCGAACGTGAACAAGAGATGATCATTAAGTTGATAGACAATAATGAAGGTCGCCCAGATGATTTGAATGGCTGCGGTATTCTCTGCTCCAATTTCCCTTGCCACCTCTGc                                                                                                                                                                                                                                           >  1:204407/1‑301 (MQ=255)`  
                                                                               `cccGCTTCCCGTTCGTCCGTCTGTTACTCAAACGAATCAAGGAGGTGTTCTGATGGGACTGTTAGATGGTGAAGCCTGGGAAAAAGAAAACCCGCCAGTACAAGCAACTGGGTGTATAGCTTGCTTAGAGAAAGATGACCGTTATCCACACACCTGTAACAAAGGAGCTAACGATATGACCGAACGTGAACAAGAGATGATCATTAAGTTGATAGACAATAATGAAGGTCGCCCAGATGATTTGAATGGCTGCGGTATTCTCTGCTCCAATGTCCCTTGCCACCTCTGCCCCGCAAATAAc                                                                                                                                                                                                                               <  1:552925/301‑1 (MQ=255)`  
                                                                                `ccGCTTCCCGTTCGTCCGTCTGTTACTCAAACGAATCAAGGAGGTGTTCTGATGGGACTGTTAGATGGTGAAGCCTGGGAAAAAGAAAACCCGCCAGTACAAGCAACTGGGTGTATAGCTTGCTTAGAGAAAGATGACCGTTATCCACACACCTGTAACAAAGGAGCTAACGATATGACCGAACGTGAACAAGAGATGATCATTAAGTTGATAGACAATAATGAAGGTCGCCCAGATGATTTGAATGGCTGCGGTATTCTCTGCTCCAATGTCCCTTGCCACCTCTGCCCCGCAAATAACg                                                                                                                                                                                                                              <  1:622353/301‑1 (MQ=255)`  
                                                                                `ccGCTTCCCGTTCGTCCGTCTGTTACTCAAACGAATCAAGGAGGTGTCCTGATGGGACTGTTAGATGGTGAAGCCTGGGAAAAAGAAAACCCGCCAGTACAAGCAACTGGGTGTATAGCTTGATTAGAGAAAGATGACCGTTATCCACACACCTGTAACAAAGGAGATAACGATATGACCGAACGTGAACAAGAGATGATCATTAAGTTGATAGACAATAATGAAGGTCGCCCAGATGATTTGAATGGCTGCGGTATTCTCTGCTCCAATGTCCCTTGCCACCTCTGCCCCGCAAATAACg                                                                                                                                                                                                                              >  1:288453/1‑301 (MQ=255)`  
                                                                                              `tccgtcTGTTACTAAAACGAATCAAGGAGGTGTTCTGATGGGACTGTTAGATGGTGAAGCCTGGGAAAAAGAAAACCCGCCAGTACAAGCAACTGGGTGTATAGCTTGCTTAGAGAAAGATGACCGTTATCCACACACCTGTAACAAAGGAGCTAACGATATGACCGAACGTGAACAAGAGATGATCATTAAGTTGATAGACAATAATGAAGGTCGCCCAGATGATTTGAATGGCTGCGGTATTCTCTGCTCCAATGTCCCTTGCCACCTCTGCCCCGCAAATAACGATCAAAAGATAAcc                                                                                                                                                                                                                <  1:143600/301‑1 (MQ=255)`  
                                                                                                 `gtcTGTTACTCAAACGAATCAAGGAGGTGTTCTGATGGGACTGTTAGATGGTGAAGCCTGGGAAAAAGAAAACCCGCCAGTACAAGCAACTGGGTGTATAGCTTGCTTAGAGAAAGATGACCGTTATCCACACACCTGTAACAAAGGAGCTAACGATATGACCGAACGTGAACAAGAGATGATCATTAAGTTGATAGACAATAATGAAGGTCGCCCAGATGATTTGAATGGCTGCGGTATTCTCTGCTCCAATGTCCCTTGCCACCTCTGCCCCGCAAATAACGATCAAAAGactgtctct                                                                                                                                                                                                             >  1:667384/1‑293 (MQ=255)`  
                                                                                                     `tttACTCAAACGAATCAGGGAGGTGTTCTGATGGGACTGTTAGATGGTGAAGCCTGGGAAAAAGAAAACCCGCCAGTACAAGCAACTGGGTGTATAGCTTGCTTAGAGAAAGATGACCGTTATCCACACACCTGTAACAAAGGAGCTAACGATATGACCGAACGTGAACAAGAGATGATCATTAAGTTGATAGACAATAATGAAGGTCGCCCAGATGATTTGAATGGCTGCGGTATTCTCTGCTCCAATGTCCCTTGCCACCTCTGCCCCGCAAATAACGATCAAAAGATAACCTTAGGTg                                                                                                                                                                                                         <  1:359936/300‑1 (MQ=255)`  
                                                                                                     `tttACTCAAACGAATCAAGGAGGTGTTCTGATGGGACTGTTAGATGGTGAAGCCTGGGAAAAAGAAAACCCGCCAGTACAAGCAACTGGGTGTATAGCTTGCTTAGAGAAAGATGACCGTTATCCACACACCTGTAACAAAGGAGCTAACGATATGACCGAACGTGAACAAGAGATGATCATTAAGTTGATAGACAATAATGAAGGTCGCCCAGATGATTTGAATGGCTGCGGTATTCTCTGCTCCAATGTCCCTTGCCACCTCTGCCCCGCAAATAACGATCAAAAGATAACCTTAGGTg                                                                                                                                                                                                         <  1:629908/300‑1 (MQ=255)`  
                                                                                                          `gtataagagacagggaggTGTTCTGAGGGGACTGTGAGATGGTGAAGCCTGGGAAAAAGAAAACCCGCCAGTACAAGCAACTGGGTGTATAGCTTGCTTAGAGAAAGATGACCGTTATCCACACACCTGTAACAAAGGAGCTAACGATATGACCGAACGTGAACAAGAGATGATCATTAAGTTGATAGACAATAATGAAGGTCGCCCAGATGATTTGAATGGCTGCGGTATTCTCTGCTCCAATGTCCCTTGCCACCTCTGCCCCGCAAATAACGATCAAAAGATAACCTTAGGTGAAATc                                                                                                                                                                                                    <  1:793976/288‑1 (MQ=255)`  
                                                                                                               `cGAATCAAGGAGGTGTTCTGATGGGACTGTTAGATGGTGAAGCCTGGGAAAAAGAAAACCCGCCAGTACAAGCAACTGGGTGTATAGCTTGCTTAGAGAAAGATGACCGTTATCCACACACCTGTAACAAAGGAGCTCACGATATGACCGAACGTGAACAAGAGATGATCATTAAGTTGATAGACAATAATGAAGGTCGCCCAGATGATTTGAATGGCTGCGGTATTCTCTGCTCCAATGTCCCTTGCCACCTCTGCCCCGCAAATAACGATCAAAAGATACCCTTAGGTGAAATCCGAGc                                                                                                                                                                                               >  1:331143/1‑301 (MQ=255)`  
                                                                                                                             `gtTCTGATGGGACTGTTAGATGGTGAAGCCTGGGAAAAAGAAAACCCGCCAGTACAAGCAACTGGGTGTATAGCTTGCTTAGAGAAAGATGACCGTTATCCACACACCTGTAACAAAGGAGCTAACGATATGACCGAACGTGAACAAGAGATGATCATTAAGTTGATAGACAATAATGAAGGTCGCCCAGATGATTTGAATGGCTGCGGTATTCTCTGCTCCAATGTCCCTTGCCACCTCTGCCCCGCAAATAACGATCAAAAGATAACCTTAGGTGAAATCCGAGCGATAGACCCACGTa                                                                                                                                                                                 >  1:740499/1‑301 (MQ=255)`  
                                                                                                                              `gcaggATGGTACTGTGAGATGGTGAGGCCTGGGAAAAAGAAAACCCGCCAGTACAAGCAACTGGGTGTATAGCTTGCTTAGAGAAAGATGACCGTTATCCACACACCTGTAACAAAGGAGCTAACGATATGACCGAACGTGAACAAGAGATGATCATTAAGTTGATAGACAATAATGAAGGTCGCCCAGATGATTTGAATGGCTGCGGTATTCTCTGCTCCAATGTCCCTTGCCACCTCTGCCCCGCAAATAACGATCAAAAGATAACCTTAGGTGAAATCCGAGCGATGGACCCACGTaa                                                                                                                                                                                <  1:100019/297‑1 (MQ=255)`  
                                                                                                                                `atccagggACTGTAAGAGGGGAAAGCCTGGAAAAAAGAAAACCCGCCAGTACAAGCAACTGGTTGTATAGCATGCTTAGAGAAAGATGACCGTTATCCACACACCTGTAACAAAGGAGCTAACGATATGACCGAACGTGAACAAGAGATGATCATTAAGTTGATAGACAATAATGAAGGTCGCCCAGATGATTTGAATGGCTGCGGTATTCTCTGCTCCAATGTCCCTTGCCACCTCTGCCCCGCAAATAACGATCAAAAGATAACCTTAGGTGAAATCCGAGCGATGGACCCACGTAAAc                                                                                                                                                                              <  1:825535/296‑1 (MQ=255)`  
                                                                                                                                      `ggACTGTTCGATGGTGAAGCCTGGGAAAAAGAAAACCCGCCAGTACAAGCAACTGGGTGTATAGCTTGCTTAGAGAAAGATGACCGTTATCCACACACCTGTAACAAAGGAGCTAACGATATGACCGAACGTGAACAAGAGATGATCATTAAGTTGATAGACAATAATGAAGGTCGCCCAGATGATTTGAATGGCTGCGGTATTCTCTGCTCCAATGTCCCTTGCCACCTCTGCCCCGCAAATAACGATCAAAAGATAACCTTAGGTGAAATCCGAGCGATGGACACACGTAAACCACATc                                                                                                                                                                        >  1:195440/1‑301 (MQ=255)`  
                                                                                                                                      `agACTGTAAGATGGAGAAGCCTGGGAAAAAGAAAACCCGCCAGTACAAGCAACTGGGTTTATAGCTTGTTTAGAGAAAAAAGACCGTTATCCACACACCTGTAACAAAAGAGCTAACGATATGACCGAACGTGAACAAGAGATGATCATTAAGTTGATAGACAATAATGAAGGTCGCCCAGATGATTTGAATGGCTGCGGTATTCTCTGCTCCAATGTCCCTTGCCACCTATGCCCCGCAAATAACGATCAAAAGATAACCTTAGGTGAAATCCGAGCGATGGACCCACGTAAACCACATc                                                                                                                                                                        <  1:566919/300‑1 (MQ=255)`  
                                                                                                                                           `gTTAGATGGTTAAGCCTGGGAAAAAGAAAACCCGCCAGTACAAGCAACTGGGTGTATAGCTTGCTTAGAGAAAGATGACCGTTATCCACACACCTGTAACAAAGGAGCTAACGATATGACCGAACGTGAACAAGAGATGATCATTAAGTTGATAGACAATAATGAAGGTCGCCCAGATGATTTGAATGGCTGCGGTATTCTCTGCTCCAATGTCCCTTGCCACCTCTGCCCCGCAAATAACGATCAACAGATAACCTTAGGTGAAATCCGAGCGATGGACCCACGTAACCCACATCTGAAt                                                                                                                                                                   >  1:130697/1‑301 (MQ=255)`  
                                                                                                                                                `aTGGTGAAGCCTGGGAAAAAGAAAACCCGCCAGTACAAGCAACTGGGTGTATAGCTTGCTTAGAGAAAGATGACCGTTATCCACACACCTGTAACAAAGGAGCTAACGATATGACCGAACGTGAACAAGAGATGATCATTAAGTTGATTGACAATAATGAAGGTCGCCCAGATGATTTGAATGGCTGCGGTATTCTCTGCTCCAATGTCCCTTGCCACCTCTGCCCCGAAAATAACGATCAAAAGATAACCTTAGGTGAAATCCGAGCGATGGACCCACGTAAACCACATCTGAATAAAcc                                                                                                                                                              <  1:732583/301‑1 (MQ=255)`  
                                                                                                                                                           `tGGGAAAAAGAAAACCCGCCAGTACAAGCAACTGGGTGTATAGCTTGCTTAGAGAAAGATGACCGTTATCCACACACCTGTAACAAAGGAGCTAACGATATGACCGAACGTGAACAAGAGATGATCATTAAGTTGATAGACAATAATGAAGGTCGCCCAGATGATTTGAATGGCTGCGGTATTCTCTGCTCCAATGTCCCTTGCCACCTCTGCCCCGCAAATAACGATCAAAAGATAACCTTAGGTGAAATCCGAGCGATGGACCCACGTAAACCACATCTGAATAAACCTGAGGTAACTc                                                                                                                                                   <  1:436617/301‑1 (MQ=255)`  
                                                                                                                                                                 `gtgtataagagacaggTACGAGCAACTGGGTGTATAGCTTGCTTAGAGAAAGATGACCGTTATCCACACACCTGTAACAAAGGAGCTAACGATATGACCGAACGTGAACAAGAGATGATCATTAAGTTGATAGACAATAATGAAGGTCGCCCAGATGATTTGAATGGCTGCGGTATTCTCTGCTCCAATGTCCCTTGCCACCTCTGCCCCGCAAATAACGAGCAAAAGATAACCTTAGGTGAAATCCGAGCGATGGACCCACGTAAACCACATCTGAATAAACCTGAGGTAACTCCTACAg                                                                                                                                             <  1:408982/286‑1 (MQ=255)`  
                                                                                                                                                                   `aGAAAACCCGCCAGTACAAGCAACTGGGTGTATAGCTTGCTTAGAGAAAGATGACCGTTATCCACACACCTGTAACAAAGGAGCTAACGATATGACCGAACGTGAACAAGAGATGATCATTAAGTTGATAGACAATAATGAAGGTCGCCCAGATGATTTGAATGGCTGCGGTATTCTCTGCTCCAATGTCCCTTGCCACCTCTGCCCCGCAAATAACGATCAAAAGATAACCTTAGGTGAAATCCTAGCGATGGACCCACGTAAACCACATCTGAATAAACCTGAGGTAACTCCTACAGAt                                                                                                                                           <  1:151491/301‑1 (MQ=255)`  
                                                                                                                                                                        `aCCCGCCAGTACAAGCAACTGGGTGTATAGCTTGCTTAGAGAAAGATGACCGTTATCCACACACCTGTAACAAAGGAGCTAACGATATGACCGAACGTGAACAAGAGATGATCATTAAGTTGATAGACAATAATGAAGGTCGCCCAGATGATTTGAATGGCTGCGGTATTCTCTGCTCCAATGTCCCTTGCCACCTCTGCCCCGCAAATAACGATCAAAAGATAACCTTAGGTGAAATCCGAGCGATGGACCCACGTAAACCACATCTGAATAACCCTGAGGTAACTCCTACAGATGACCa                                                                                                                                      >  1:472807/1‑301 (MQ=255)`  
                                                                                                                                                                             `ccAGTACAAGCAACTGGGTGTATAGCTTGCTTAGAGAAAGATGACCGTTATCCACACACCTGTAACAAAGGAGCTAACGATAGGACCGAACGTGAACAAGAGATGATCATTAAGATGATAGACAATAATGAAGGTCGCCCAGATGATTTGAATGGCTGCGGTATTCTCTGCTCCAATGTCCCTTGCCACCTCTGCCCCGCAAATAACGATCAAAAGATAACCTTAGGTGAAATCCGAGCGATGGACCCACGTGAACCACATCTGAATAAACCTGAGGTAACTCCTACAGATGACCAGccat                                                                                                                                 >  1:503620/1‑299 (MQ=255)`  
                                                                                                                                                                              `cAGTACAAGCAACTGGGTGTATAGCTTGCTTAGAGAAAGATGACCGTTATCCACACACCTGTAACAAAGGAGCTAACGATATGACCGAACGTGAACAAGAGATGATCATTAAGTTGATAGACAATAATGAAGGTCGCCCAGATGATTTGAATGGCTGCGGTATTCTCTGCTCCAATGTCCCTTGCCACCTCTGCCCCGCAAATAACGATCAAAAGATAACCTTAGGTGAAATCCGAGCGATGGACCCACGTAAACCACATCTGAATAAACCTGAGGTAACTCCTACAGATGACCAGCCTTc                                                                                                                                <  1:91814/301‑1 (MQ=255)`  
                                                                                                                                                                                 `tACCAGCAACTGGGTGTATAGCTTGCTTAGAGAAAGATGACCGTTATCCACACACCTGTAACAAAGGAGCTAACGATATGACCGAACGTGAACAAGAGATGATCATTAAGTTGATAGACAATAATGAAGGTCGCCCAGATGATTTGAATGGCTGCGGTATTCTCTGCTCCAATGTCCCTTGCCACCTCTGCCCCGCAAATAACGATCAAAAGATAACCTTAGGTGAAATCCGAGCGATGGACCCACGTAAACCACATCTGAATAAACCTGAGGTAACTCCTACAGATGACCAGCCTTCCGc                                                                                                                             <  1:465180/301‑1 (MQ=255)`  
                                                                                                                                                                                   `cAAGCAACTGGGTGTATAGCTTGCTTAGAGAAAGATGACCGTTATCCACACACCTGTAACAAAGGAGCTAACGATATGACCGAACGTGAACAAGAGATGATCATTAAGTTGATAGACAATAATGAAGGTCGCCCAGATGATTTGAATGGCTGCGGTATTCTCTGCTCCAATGTCCCTTGCCACCTCTGCCCCGCAAATAACGATCAAAAGATAACCTTAGGTGAAATCCGAGCGATGGACCCACGTAAACCACATCTGAATAAACCTGAGGTAACTCCTACAGATGACCAGCCTTCCGCTg                                                                                                                           >  1:787663/1‑301 (MQ=255)`  
                                                                                                                                                                                       `cAACTGGGTGTATAGCTTGCTTAGAGAAAGATGACCGTTATCCACACACCTGTAACAAAGGAGCTAACGATATGACCGAACGTGAACAAGAGATGATCATTAAGTTGATAGACAATAATGAAGGTCGCCCAGATGATTTGAATGGCTGCGGTATTCTCTGCTCCAATGTCCCTTGCCACCTCTGCCCCGCAAATAACGATCAAAAGATAACCTTAGGTGAAATCCGAGCGATGGACCAACGTAAACCACATCTGAATAAACCTGAGGTAACTCCTAAAGTTGACCAGCCTTCCGCTGAGAc                                                                                                                       >  1:682299/1‑301 (MQ=255)`  
                                                                                                                                                                                             `ggTGTATAGCTTGCTTAGAGAAAGATGACCGTTATCCACACACCTGTAACAAAGGAGCTAACGATATGACCGAACGTGAACAAGAGATGATCATTAAGTTGATAGACAATAATGAAGGTCGCCCAGATGATTTGAATGGCTGCGGTATTCTCTGCTCCAATGTCCCTTGCCACCTCTGCCCCGCAAATAACGATCAAAAGATAACCTTAGGTGAAATCCGAGCGATGGACCCACGTAAAACACATCTGAATAAACCTGAGGTAACTCCTACAGATGACCAGCCTTCCGCTGAGACAATCGa                                                                                                                 >  1:318649/1‑301 (MQ=255)`  
                                                                                                                                                                                              `gtgtATAGCTTGCTTAGAGAAAGATGACCGTTATCCACACACCTGTAACAAAGGAGCTAACGATATGACCGAACGTGAACAAGAGATGATCATTAAGTTGATAGACAATAATGAAGGTCGCCCAGATGATTTGAATGGCTGCGGTATTCTCTGCTCCAATGTCCCTTGCCACCTCTGCCCCGCAAATAACGATCAAAAGATAACCTTAGGTGAAATCCGAGCGATGGACCCACGTAAACCACATCTGAATAAACCTGAGGTAACTCCTACAGATGACCAGCCTTCCGCTGAGACAATCGaa                                                                                                                >  1:689303/1‑301 (MQ=255)`  
                                                                                                                                                                                              `gtgtATAGCTTGCTTAGAGAAAGATGACCGTTATCCAAAAACCTGTAACAAAGGAGCTAACGATATGACCGAACGTGAACAAGAGATGATCATTAAGTTGATAGACAATAATGAAGGTCGCCCAGATGATTTGAAGGGCTGCGGTATTCTCTGCTCCAATGTCCCTTGCCACCTCTGCCCCGCAAATAACGATCAAAAGATAACCTTAGGTGAAATCCGAGCGATGGACCCACGTAAACCACATCTGAATAAACCTGAGGTAACTCCTACAGATGACCAGCCTTCCGCTGAGACAATCGaa                                                                                                                <  1:304473/301‑1 (MQ=255)`  
                                                                                                                                                                                                    `aGCTTGCTTAGAGAAAGATGACCGTTATCCACACACCTGTAACAAAGGAGCTAACGATATGACCGAACGTGAACAAGAGATGATCATTAAGTTGATAGACAATAATGAAGGTCGCCCAGATGATTTGAATGGCTGCGGTATTCTCTGCTCCAATGTCCCTTGCCACCTCTGCCCCGCAAATAACGATCAAAAGATAACCTTAGGTGAAATCCGAGCGATGGACCCACGTAAACCACATCTGAATAAACCTGAGGTAACTCCTACAGATGACCAGCCTTCCGCTGAGAcctgtctcttatac                                                                                                          >  1:644733/1‑288 (MQ=255)`  
                                                                                                                                                                                                              `gagaAAGATGACCGTTATCCACACACCTGTAACAAAGGAGCTAACGATATGACCGAACGTGAACAAGAGATGATCATTAAGTTGATAGACAATAATGAAGGTCGCCCAGATGATTTGAATGGCTGCGGTATTCTCTGCTCCAATGTCCCTTGCCACCTCTGCCCCGCAAATAACGATCAAAAGATAACCTTAGGTGAAATCCGAGCGATGGACCCACGTAAACCACATCTGAATAAACCTGAGGTAACTCCTACAGATGACCAGCCTTCCGcctgtctcttatacacatctccgagcccac                                                                                                >  1:810917/1‑272 (MQ=255)`  
                                                                                                                                                                                                                   `agagGACCGTTATCCACAAACCTGTAACAAAGGAGCTAACGATATGACCGAACGTGAACAAGAGATGATCATTAAGTTGATAGACAATAAGGAAGGTCGCCCAGATGATTTGAATGGCTGCGGTATTCTCTGCTCCAATGTCCCTTGCCACCTCTGCCCCGCAAATAACGATCAAAAGATAACCTTAGGTGAAATCCGAGCGATGGACCCACGTAAACCACATCTGAATAAACCTGAGGTAACTCCTACAGATGACCAGCCTTCCGCTGAGACAATCGAAGGTGTCACTAAGCCTTCCCAc                                                                                           <  1:243205/301‑1 (MQ=255)`  
                                                                                                                                                                                                                      `tGACCGTTATCCACACACCTGTAACAAATGAGCTAACGATATGACCGAACGTGAACAAGAGATGATCATTAAGTTGATAGACAATAATGAAGGTCGCCCAGATGATTTGAATGGCTGCGGTATTCTCTGCTCCAATGTCCCTTGCCACCTCTGCCCCGCAAATAACGATCAAACGATAACCTTAGGTGAAATCCTAGCGATGGACCCACGTAAACCACATCTGAATAAACCTGAGGTAACTCCTACAGATGACCAGCCTTCCGCTGAGACAATCGAAGGTGTCACTAAGCCTTCCCACTAc                                                                                        <  1:428626/301‑1 (MQ=255)`  
                                                                                                                                                                                                                         `ccGTTATCCACACACCTGTAACAAAGGAGCTAACGATATGACCGAACGTGAACAAGAGATGATCATTAAGTTGATAGACAATAATGAAGGTCGCCCAGATGATTTGAATGGCTGCGGTATTCTCTGCTCCAATGTCCCTTGCCACCTCTGCCACGCAAATAACGATCAAAAGATAACCTTAGGTGAAATCCGAGCGATGGCCCCACGTCAACCACATCTGAATCAACCTGAGGTAACTCCTACAGATGACCAGCCTTCCGCTGCGACAATCCAAGGTTTCACTAAGCCTTCCCACTACAtt                                                                                     >  1:380913/1‑300 (MQ=255)`  
                                                                                                                                                                                                                         `ccGTTATCCACACACCTGTAACAAAGGAGCTAACGATATGACCGAACGTGAACAAGAGATGATCATTAAGTTGATAGACAATAATGAAGGTCGCCCAGATGATTTGAATGGCTGCGGTATTCTCTGCTCCAATGTCCCTTGCCACCTCTGCCACGCAAATAACGATCAAAAGATAACCTTAGGTGAAATCCGAGCGATGGACCCACGTAAACCACATCTGAATAAACCTGAGGTAACTCCTACAGATGACCAGCCTTCCGCTGAGACAATCGAAGGGGTCACTAAGCCTTCCCACTAcctt                                                                                     >  1:296635/1‑298 (MQ=255)`  
                                                                                                                                                                                                                         `aggtaaTCCAAACACCTGTAACAAAGGGGCTAACGATATGACCGAACGTGAACAAGAGATGATCATTAAGTTGATAGACAATAATGAAGGTCGCCCAGATGATTTGAATGGCTGCGGTATTCTCTGCTCCAATGTCCCTTGCCACCTCTGCCCCGCAAATAACGATCAAAAGATAACCTTAGGTGAAATCCGAGCGATGGACCCACGTAAACCACATCTGAATAAACCTGAGGTAACTCCTACAGATGACCAGCCTTCCGCTGAGACAATCGAAGGTGTCACTAAGCCTTCCCACTACATg                                                                                     <  1:52021/296‑1 (MQ=255)`  
                                                                                                                                                                                                                            `ttATCCACACACCTGTAACAAAGGAGCTAACGATATGACCGAACGTGAACAAGAGATGATCATTAAGTTGATAGACAATAATGAAGGTCGCCCAGATGATTTGAATGGCTGCGGTATTCTCTGCTCCAATGTCCCTTGCCACCTCTGCCCCGCAAATAACGATCAAAAGATAACCTTAGGTGAAATCCGAGCGATGGACCCACGTAAACCACATCTGAATAAACCTAAGGTAACTCCTACAGATGACCAGCCTTCCGCTGAGACAATCGAAGGTGTCACTAAGCCTTCCCACTACATGCTg                                                                                  <  1:696520/301‑1 (MQ=255)`  
                                                                                                                                                                                                                                 `cacacaCCTGTAACAAAGGAGCTAACGATATGACCGAACGTGAACAAGAGATGATCATTAAGTTGATAGACAATAATGAAGGTCGCCCAGATGATTTGAATGGCTGCGGTATTCTCTGCTCCAATGTCCCTTGCCACCTCTGCCCCGCAAATAACGATCAAAAGATAACCTTAGGTGAAATCCGAGCGATGGACCCACGTAAACCACATCTGAATAAACCTGAGGTAACTCCTACAGATGACCAGCCTTCCGCTGAGACAATCGAAGGTGTCACTAAGCCTTCCCACTACATGCTGTTTga                                                                             >  1:266834/1‑301 (MQ=255)`  
                                                                                                                                                                                                                                  `acacacCTGTAACAAAGGAGCTAACGATATGACCGAACGTGAACAAGAGATGATCATTAAGTTGATAGACAATAATGAAGGTCGCCCAGATGATTTGAATGGCTGCGGTATTCTCTGCTCCAATGTCCCTTGCCACCTCTGCCCCGCAAATAACGATCAAAAGATAACCTTAGGTGAAATCCGAGCGATGGACCCACGTAAACCACATCTGAATAAACCTGAGGTAACTCCTACAGATGACCAGCCTTCCGCTGAGACAATCGAAGGTGTCACTAAGCCTTCCCACTACATGCTGTTTgac                                                                            <  1:544145/301‑1 (MQ=255)`  
                                                                                                                                                                                                                                       `ccTGTAACAAAGGAGCTAACGATATGACCGAACGTGAACAAGAGATGATCATTAAGTTGATAGACAATAATGAAGGTCGCCCAGATGATTTGAATGGCTGCGGTATTCTCTGCTCCAATGTCCCTTGCCACCTCTGCCCCGCAAATAACGATCAAAAGATAACCTTAGGTGAAATCCGAGCGATGGACCCACGTAAACCACATCTGAATAAACCTGAGGTAACTCCTACAGATGACCAGCCTTCCGCTGATACAATCGAAGGTGTCACTAAGCCTTCCCACTACATGCTGTTTGACGACac                                                                       >  1:818107/1‑300 (MQ=255)`  
                                                                                                                                                                                                                                           `tAACAAAGGAGCTAACGATATGACCGAACGTGAACAAGAGATGATCATTAAGTTTATAGACAATAATGAAGGTCGCCCAGATGATTTGAATGGCTGCGGTATTCTCTGCTCCAATGTCCCTTGCCACCTCTGCCCCGCAAATAACGATCAAAAGATAACCTTAGGTGAAATCCGAGCGATGGACCCACGTAAACCACATCTGAATAAACCTGAGGTAACTCCTACAGATGACCAGCCTTCCGCTGAGACAATCGAAGGTGTCACTAAGCCTTCCCACTACATGCTGTTTGACGACAtctgt                                                                   >  1:228377/1‑297 (MQ=255)`  
                                                                                                                                                                                                                                           `tAACAAAGGAGCTAACGATATGACCGAACGTGAACAAGAGATGATCATTAAGTCGATAGACAATAATGAAGGTCGCCCAGATGATTTGAATGGCTGCGGTATTCTCTGCTCCAATGTCCCTTGCCACCTCTGCCCCGCAAATAACGATCAAAAGATAACCTTAGGTGAAATCCGAGCGATGGACCCACGTAAACCACATCTGAATAAACCTGAGGTAACTCCTACAGATGACCAGCCTTCCGCTGAGACAATCGAAGGTGTCACTAAGCCTTCCCACTACATGCTGTCTgtctcttataca                                                                   >  1:171384/1‑290 (MQ=255)`  
                                                                                                                                                                                                                                             `ccAAAGGAGCTAACGATATGACCGAACGTGAACAAGAGATGATCATTAAGTTGATAGACAATAATGAAGGTCGCCCAGATGATTTGAATGGCTGCGGTATTCTCTGCTCCAATGTCCCTTGCCACCTCTGCCCCGCAAATAACGATCAAAAGATAACCTTAGGTGAAATCCGAGCGATGGACCCACGTAAACCACATCTGAATAAACCTGAGGTAACTCCTACAGATGACCAGCCTTCCGCTGAGACAATCGAAGGTGTCACTAAGCCTTCCCACTACATGCTGTTTGACGACATTGAGGc                                                                 <  1:219046/300‑1 (MQ=255)`  
                                                                                                                                                                                                                                             `aCAAAGGAGCTAGCGATATGACCGAACGTGAACAAGAGATGATCATTAAGTTGATAGACAATAATGAAGGTCGCCCAGATGATTTGAATGGCTGCGGTATTCTCTGCTCCAATGTCCCTTGCCACCTCTGCCCCGCAAATAACGATCAAAAGATAACCTTAGGTGAAATCCGAGCGATGGACCCACGTAAACCACATCTGAATAAACCTGAGGTAACTCCTACAGATGACCAGCCTTTCGCTGAGACAATCGAAGGTGTCACTAAGCCTTCCCACTACATGCTGTTTGACGACATTGAGGc                                                                 <  1:116304/301‑1 (MQ=255)`  
                                                                                                                                                                                                                                             `aCAAAGGAGCTAACGATATGACCGAACGTGAACAAGAGATGATCATTAAGTTGATAGACAATAATGAAGGTCGCCCAGATGATTTGAATGGCTGCGGTATTCTCTGCTCCAATGTCCCTTGCCACCTCTGCCCCGCAAATAACGATCAAAAGATAACCTTAGGTGAAATCCGAGCGATGGACCCACGTAAACCACATCTGAATAAACCTGAGGTAACTCCTACAGATGACCAGCCTTCCGCTGAGACAATCGAAGGTGTCACTAAGCCTTCCCACTACATGCTGTTTGACGACATTGAGGc                                                                 <  1:582990/301‑1 (MQ=255)`  
                                                                                                                                                                                                                                               `aaaGGAGCTAACGATATGACCGAACGTGAACAAGAGATGATCATTAAGTTGATAGACAATAATGAAGGTCGCCCAGATGATTTGAATGGCTTCGGTATTCTCTGCTCCAATGTCCCTTTCCACCTCTG‑CCCGCAAATAACGATCAAAAGATAACCTTAGGTGAAATCCGAGCGATGGACCCACGTAAACCACATCTGAATAAACCTGAGGTAACTCCTACAGATGACCAGCCTTCCGCTGAGACAATCGAATGTGTCACTAAGCCTTCCCACTCCATGCTGTTTGACGACATTGAGGCTAt                                                              >  1:637500/1‑301 (MQ=255)`  
                                                                                                                                                                                                                                                  `ggAGCTAACGATATGACCGAACGTGAACAAGAGATGATCATTAAGTTGATAGACAATAATGAAGGTCGCCCAGATGATTTGAATGGCTGCGGTATTCTCTGCTCCAATGTCCCTTGCCACCTCTGCCCCGCAAATAACGATCAAAAGATAACCTTAGGTGAAATCCGAGCGATGGACCCACGTAAACCACATCTGAATAAACCTGAGGTAACTCCTACAGATGACCAGCCTTCCGCTGAGACAATCGAAGGTGTCACTAAGCCTTCCCACTACATGCTGTTTGACGACATTGAGGCTATCg                                                            >  1:274074/1‑301 (MQ=255)`  
                                                                                                                                                                                                                                                  `ggAGCTAACGATATGACCGAACGTGAACAAGAGATGATCATTAAGTTGATAGACAATAATGAAGGTCGCCCAGATGATTTGAATGGCTGCGGTATTCTCTGCTCCAATGTCCCTTGCCACCTCTGCCCCGCAAATAACGATCAAAAGATAACCTTAGGTGAAATCCGAGCGATGGACCCACGTAAACCACATCTGAATAAACCTGAGGTAACTCCTACAGATGACCAGCCTTCCGCTGAGACAATCGAAGGTGTCACTAAGCCTTCCAACTACATGCTGTTTGACGACATTGAGGcctgtc                                                            >  1:258337/1‑296 (MQ=255)`  
                                                                                                                                                                                                                                                  `ggAGCTAACGATATGACCGAACGTGAACAAGAGATGATCATTAAGTTGATAGACAATAATGAAGGTCGCCCAGATGATTTGAATGGCTGCGGTATTCTCTGCTCCAATGTCCCTTGCCACCTCTGCCCCGCAAATAACGATCAAAAGATAACCTTAGGTGAAATCCGAGAGATGGACCCACGTAAACCACATCTGAATAAACCTGAGGTAACTCCTACAGATGACCAGCCTTCCGCTGAGACAATCGAAGGTGTCACTAAGCCTTCCCACTACATGCTGTTTGACGACATTGAGGCTATCg                                                            >  1:755597/1‑301 (MQ=255)`  
                                                                                                                                                                                                                                                   `gAGCTAACGATATGACCGAACGTGAACAAGAGATGATCATTAAGTTGATAGACAATAATGAAGGTCGCCCAGATGATTTGAATGGCTGCGGTATTCTCTGCTCCAATGTCCCTTGCCACCTCTGCCCCGCAAATAACGATCAAAAGATAACCTTAGGTGAAATCCGAGCGATGGACCCACGTAAACCACATCTGAATAAACCTGAGGTAACTCCTACAGATGACCAGCCTTCCGCTGAGACAATCGAAGGTGTCACTAAGCCTTCCCACTACATGCTGTTTGACGACATTGAGGCTATCGa                                                           >  1:458850/1‑301 (MQ=255)`  
                                                                                                                                                                                                                                                     `gCTAACGATATGACCGAACGTGAACAAAAGATGATCATTAAGTTGATAGACAATAATGAAGGTCGCCCAGATGATTTGAATGGCTGCGGTATTCTCTGCTCCAATGTCCCTTGCCACCTCTGCCCCGCAAATAACGATCAAAAGATAACCTTAGGTGAAATCCGAGCGATGGACCCACGTAAACCACATCTGAATAAACCTGAGGTAACTCCTACAGATGACCAGCCTTCCGATGAGACAATCGAAGGTGTCACTAAGCCTTCCCACTACATGCTGTTTGACGACATTGAGGCTATCGAAg                                                         <  1:281303/301‑1 (MQ=255)`  
                                                                                                                                                                                                                                                          `cGATATGACCGAACGTGAACAAGAGATGATCATTAAGTTGATAGACAATAATGAAGGTCGCCCAGATGATTTGAATGGCTGCGGTATTCTCTGCTCCAATGTCCCTTGCCACCTCTGCCCCGCAAATAACGATCAAAAGATAACCTTAGGTGAAATCCGAGCGATGGACCCACGTAAACCACATCTGAATAAACCTGAGGTAACTCCTACAGATGACCAGCCTTCCGCTGAGACAATCGAAGGTGTCACTAAGCCTTCCCACTACATGCTGTTTGACGACATTGAGGCTATCGAAGTGAtt                                                    >  1:56001/1‑301 (MQ=255)`  
                                                                                                                                                                                                                                                            `atatGACCGAACGTGAACAAGAGATGATCATTAAGTTGATAGACAATAATGAAGGTCGCCCAGATGATTTGAATGGCTGCGGTATTCTCTGCTCCAATGTCCCTTGCCACCTCTGCCCCGCAAATAACGATCAAAAGATAACCTTAGGTGAAATCCGAGCGATGGACCCACGTAAACCACATCTGAATAAACCTGAGGTAACTCCTACAGATGACCAGCCTTCCGCTGAGACAATCGAAGGTGTCACTAAGCCTTCCCACTACATGCTGTTTgacgacctgtctcttatacacatctccga                                                  >  1:444652/1‑278 (MQ=255)`  
                                                                                                                                                                                                                                                             `tatGACCGAACGTGAACAAGAGATGATCATTAAGTTGATAGACAATAATGAAGGTCGCCCAGATGATTTGAATGGCTGCGGTATTCTCTGCTCCAATGTCCCTTGCCACCTCTGCCCCGCAAATAACGATCAAAAGATAACCTTAGGTGAAATCCGAGCGATGGACCCACGTAAACCACATCTGAATAAACCTGAGGTAACTCCTACAGATGACCAGCCTTCCGCTGAGACAATCGAAGGTGTCACTAAGCCTTCCCACTACATGCTGTTTGACGACATTGAGGCTATCGAAGTGATTGCt                                                 <  1:801365/301‑1 (MQ=255)`  
                                                                                                                                                                                                                                                                     `aaCGTGAACAATAGATGATCATTAAGTTGATAGACAATAATGAAGGTCGCCCAGATGATTTGAATGGCTGCGGTATTCTCTGCTCCAATGTCCCTTGCCACCTCTGCCCCGCAAATAACGATCAAAAGATAACCTTAGGTGAAATCCGAGCGATGGACCCACGTAAACCACATCTGAATAAACCTGAGGTAACTCCTACAGATGACCAGCCTTCCGCTGAGACAATCGAAGGTGTCACTAAGCCTTCCCACTACATGCTGTTTGACGACATTGAGGCTATCCAAGTTATTGCTCGTTCAAt                                         >  1:402219/1‑301 (MQ=255)`  
                                                                                                                                                                                                                                                                     `aaCGTGAACAAGAGATGATCATTAAGTTGATAGACAATAATGAAGGTCGCCCAGATGATTTGAATGGCTGCGGTATTCTCTGCTCCAATGTCCCTTGCCACCTCTGCCCCGCAAATAACGATCAAAAGATAACCTTAGGTGAAATCCGAGCGATGGACCCACGTAAACCACATCTGAATAAACCTGAGGTAACTCCTACAGATGACCAGCCTTCCGCTGAGACAATCGAAGGTGTCACTAAGCCTTCCCACTACATGCTGTTTGACGACATTGAGGCTATCGAAGTGATTGCTCGTTCAAt                                         >  1:136991/1‑301 (MQ=255)`  
                                                                                                                                                                                                                                                                        `gTGAACAAGAGATGATCATTAAGTTGATAGACAATAATGAAGGTCGCCCAGATGATTTGAATGGCTGCGGTATTCTCTGCTCCAATGTCCCTTGCCACCTCTGCCCCGCAAATAACGATCAAAAGATAACCTTAGGTGAAATCCGAGCGATGGACCCACGTAAACCACATCTGAATAAACCTGAGGTAACTCCTACAGATGACCAGCCTTCCGCTGAGACAATCGAAGGTGTCACTAAGCCTTCCCACTACATGCTGTTTGACGACATTGAGGCTATCGAAGTGATTGCTCGTTCAATGAc                                      <  1:236675/301‑1 (MQ=255)`  
                                                                                                                                                                                                                                                                               `agagATGATCATTAAGTTGATAGACAATAATGAAGGTCGCCCAGATGATTTGAATGGCTGCGGTATTCTCTGCTCCAATGTCCCTTGCCACCTCTGCCCCGCAAATAACGATCAAAAGATAACCTTAGGTGAAATCCGAGCGATGGACCCACGTAAACCACATCTGAATAAACCTGAGGTAACTCCTACAGATGACCAGCCTTCCGCTGAGACAATCGAAGGTGTCACTAAGCCTTCCCACTACATGCTGTTTGACGACATTGAGGATATCGAAGTGATTGCTCGTTCAATGACCGTTGAg                               >  1:180963/1‑301 (MQ=255)`  
                                                                                                                                                                                                                                                                                `gagaTGATCATTAAGTTGATAGACAATAATGAAGGTCGCCCAGATGATTTGAATGGCTGCGGTATTCTCTGCTCCAATGTCCCTTGCCACCTCTGCCCCGCAAATAACGATCAAAAGATAACCTTAGGTGAAATCCGAGCGATGGACCCACGTAAACCACATCTGAATAAACCTGAGGTAACTCCTACAGATGACCAGCCTTCCGCTGAGACAATCGAAGGTGTCACTAAGCCTTCCCACTACATGCTGTTTGACGACATTGAGGCTATCGAAGTGATTGCTCGTTCAATGACCGTTGAGc                              >  1:606887/1‑301 (MQ=255)`  
                                                                                                                                                                                                                                                                                       `tCATTAAGTTGATAGACAATAATGAAGGTCGCCCAGATGATTTGAATGGCTGCGGTATTCTCTGCTCCAATGTCCCTTGCCACCTCTGCCCCGCAAATAACGATCAAAAGATAACCTTAGGTGAAATCCGAGCGATGGACCCACGTAAACCACATCTGAATAAACCTGAGGTAACTCCTACAGATGACCAGCCTTCCGCTGAGACAATCGAAGGTGTCACTAAGCCTTCCCACTACATGCTGTTTGACGACATTGAGGCTATCGAAGTGTTTGCTCGTTCAATGCCCGTTGAGCAGTTCaa                       >  1:324989/1‑301 (MQ=255)`  
                                                                                                                                                                                                                                                                                       `tCATTAAGTTGATAGACAATAATGAAGGTCGCCCAGATGATTTGAATGGCTGCGGTATTCTCTGCTCCAATGTCCCTTGCCACCTCTGCCCCGCAAATAACGATCAAAAGATAACCTTAGGTGAAATCCGAGCGATGGACCCACGTAAACCACATCTGAATAAACCTGAGGTAACTCCTACAGATGACCAGCCTTCCGCTGAGACAATCGAAGGTGTCACTAAGCCTTCCCACTACATGCTGTTTGACGACATTGAGGCTATCGAAGTGATTGCTCGTTCAATGACCGTTGAGCAGTTcct                       >  1:311825/1‑299 (MQ=255)`  
                                                                                                                                                                                                                                                                                       `tCATTAAGTTGATAGACAATAATGAAGGTCGCCCAGATGATTTGAATGGCTGCGGTATTCTCTGCTCCAATGTCCCTTGCCACCTCTGCCCCGCAAATAACGATCAAAAGATAACCTTAGGTGAAATCCGAGCGATGGACCCACGTAAACCACATCTGAATAAACCTGAGGTAACTCCTACAGATGACCAGCCTTCCGCTGAGACAATCGAAGGTGTCACTAAGCCTTCCCACTACATGCTGTTTGACGACATTGAGGCTATCGAAGTGATTGCTCGTTCAATGACCGTTGAGCAGTTCaa                       >  1:574583/1‑301 (MQ=255)`  
                                                                                                                                                                                                                                                                                          `ttAAGTTGATATACAATAATGAAGGTCGCCAAGATGATTTGAATGGCTGCGGTATTCTCTGCTCCAATGTCCCTTGCCACCTCTGCCCCGCAAATAACGATCAAAAGATAACCTTAGGTGAAATCCGAGCGATGGACCCACGTAAACCACATCTGAATAAACCTGAGGTAACTCCTACAGATGACCAGCCTTCCGCTGAGACAATCGAAGGTGTCACTAAGCCTTCCCACTACATGCTGTTTGACGACATTGAGGCTATCGAAGTGATTGCTCGTTCAATGACCGTTGAGCAGTTCAAggg                    <  1:590762/301‑1 (MQ=255)`  
                                                                                                                                                                                                                                                                                          `ttAAGTTGATAGACAATAATGAATGTCGCCAAGAGGATTTGAATGGCTGCGGTATTCTCTGCTCCAATGTACCTTGCCACCTCTGACCCGCAAATAACGATCAAAAGATAACCTTAGGTGAAATCCGAGCGATGGACCCACGTAAACCACATCTGAATAAACCTGAGGTAACTCCTACAGATGAACAGCCTTCCGCTGAGACAATCGAAGGTGTCACTAAGCCTTCCCACTACATGCTGTTTGACGACATTGAGGCTATCGAAGTGATTGCTCGTTCAATGACCGTTGAGCAGTTCAAggg                    <  1:749273/301‑1 (MQ=255)`  
                                                                                                                                                                                                                                                                                          `ttAAGTTGATAGACAATAATGAAGGTCGCCCAGATGATTTGAATGGCTGCGGTATTCTCTGCTCCAATGTCCCTTGCCACCTCTGCCCCGCAAATAACGATCAAAAGATAACCTTAGGTGAAATCCGAGCGATGGACCCACGTAAACCACATCTGAATAAACCTGAGGTAACTCCTACAGATGACCAGCCTTCCGCTGAGACAATCGAAGGTGTCACTAAGCCTTCCCACTACATGCTGTTTGACGACATTGAGGCTATCGAAGTGATTGCTCGTTCAATGACCGTTGAGCAGTTCAAggg                    <  1:535589/301‑1 (MQ=255)`  
                                                                                                                                                                                                                                                                                          `ttAAGTTGATAGACAATAATGAAGGTCGCCCAGATGATTTGAATGACTGCGGTATTCTCTGCTCCAATGTCCCTTGCCACCTCTGCCCCGCAAATAACGATCAAAAGATAACCTTAGGTGAAATCCGAGCGATGGACACACGTAAACCACATCTGAATAAACCTGAGGTAACTCATACAGATGACCAGCCTTCCGCTGAGATAATCGAAGGTGTCACTAAGCCTTCCCACTACATGCTGTTTGACGACATTGAGGCTATCGAAGTGATTGCTCGTTCAATGACCGTTGAGCAGTTCAAggg                    <  1:495881/301‑1 (MQ=255)`  
                                                                                                                                                                                                                                                                                          `gtataagagacagcAATAATGAAGGTCGCCCAGATGATTTGAATGGCTGCGGTATTCTCTGCTCCAATGTCCCTTGCCACCTCTGCCCCGCAAATAACGATCAAAAGATAACCTTAGGTGAAATCCGAGCGATGGACCCACGTAAACCACATCTGAATAAACCTGAGGTAACTCCTACAGATGACCAGCCTTCCGCTGAGACAATCGAAGGTGTCACTAAGCCTTCCCACTACATGCTGTTTGACGACATTGAGGCTATCGAAGTGATTGCTCGTTCAATGACCGTTGAGCAGTTCAAggg                    <  1:416257/288‑1 (MQ=255)`  
                                                                                                                                                                                                                                                                                            `aggTTGATAGACAATAATGAAGGTCGCCCAGATGATTTGAATGGCTGCGGTATTCTCTGCTCCAATGTCCCTTGCCACCTCTGCCCCGCAAATAACGATCAAAAGATAACCTTAGGTGAAATCCGAGCGATGGACCCACGTAAACCACATCTGAATAAACCTGAGGTAACTCCTACAGATGACCAGCCTTCCGCTGAGACAATCGAAGGTGTCACTAAGCCTTCCCACTACATGCTGTTTGACGACATTGAGGCTATCGAAGTGATTGCTCGTTCAATGACCGTTGAGCAGTTCAAGGGAt                  <  1:421301/299‑1 (MQ=255)`  
                                                                                                                                                                                                                                                                                            `aaGTTGATAGACAATAATGAAGGTCGCCCAGATGATTTGAATGGCTGCGGTATTCTCTGCTCCAATGTCCCTTGCCACCTCTGCCCCGCAAATAACGATCAAAAGATAACCTTAGGTGAAATCCGAGCGATGGACCCACGTAAACCACATCTGAATAAACCTGAGGTAACTCCTACAGATGACCAGCCTTCCGCTGAGACAATCGAAGGTGTCACTAAGCCTTCCCACTACATGCTGTTTGACGACATTGAGGCTATCGAAGTGATTGCTCGTTCAATGACCGTTGAGCAGTTCAAGGGAt                  >  1:157410/1‑301 (MQ=255)`  
                                                                                                                                                                                                                                                                                              `ataagagacagataatGAAGGTCGCCCAGATGATTTGAATGGCTGCGGTATTCTCTGCTCCAATGTCCCTTGCCACCTATGCCCCGCAAATAACGATCAAAAGATAACCTTAGGTGAAATCCGAGCGATGGACCCACGTAAACCACATCTGAATAAACCTGAGGTAACTCCTACAGATGACCAGCCTTCCGCTGAGACAATCGAAGGTGTCACTAAGCCTTCCCACTACATGCTGTTTGACGACATTGAGGCTATCGAAGTGATTGCTCGTTCAATGACCGTTGAGCAGTTCAAGGGATAc                <  1:375175/290‑1 (MQ=255)`  
                                                                                                                                                                                                                                                                                               `ttGATAGACAATAATGAAGGTCGCCCAGATGATTTGAATGGCTGCGGTATTCTCTGCTCCAATGTCCCTTGCCACCTCTGCCCCGCAAATAACGATCAAAAGATAACCTTAGGTGAAATCCGAGCGATGGACCCACGTAAACCACATCTGAATAAACCTGAGGTAACTCCTACAGATGACCAGCCTTCCGCTGAGACAATCGAAGGTGTCACTAAGCCTTCCCACTACATGCTGTTTGACGACATTGAGGCTATCGAAGTGATTGCTCGTTCAATGACCGTTGAGCAGTTCAAGGGATaat               >  1:658539/1‑299 (MQ=255)`  
                                                                                                                                                                                                                                                                                                       `cAATAATGAAGGTCGCCCATATGATTTGAATGGCTGCGGTATTCTCTGCTCCAATGTCCCTTGCCACCTCTGCCCCGCAAATAACGATCAAAAGATAACCTTAGGTGAAATCCGAGCGATGGACCCACGTAAACCACATCTGAATAAACCTGAGGTAACTCCTACAGATGACCAGCCTTCCGCTGAGACAATCGAAGGTGTCACTAAGCCTTCCCACTACATGCTGTTTGACGACATTGAGGCTATCGAAGTGATTGCTCGTTCAATGACCGTTGAGCAGTTCAAGGGATACTGCTTCGGt       <  1:211837/301‑1 (MQ=255)`  
                                                                                                                                                                                                                                                                                                       `cAATAATGAAGGTCGCCCAGATGATTTGAATGGCTGCGGTATTCTCTGCTCCAATGTCCCTTGCCACCTCTGCCCCGCAAATAACGATCAAAAGATAACCTTAGGTGAAATCCGAGCGATGGACCCACGTAAACCACATCTGAATAAACCTGAGGTAACTCCTACAGATGACCAGCCTTCCGCTGAGACAATCGAAGGTGTCACTAAGCCTTCCCACTACATGCTGTTTGACGACATTGAGGCTATCGAAGTGATTGCTCGTTCAATGACCGTTGAGCAGACAAAGGGATACTGCTTCggg       >  1:614459/1‑300 (MQ=255)`  
                                                                                                                                                                                                                                                                                                        `cagaatGAAGGTCGCCCAGATGATTTGAATGGCTGCGGTATTCTCTGCTCCAATGTCCCTTGCCACCTCTGCCCCGCAAATAACGATCAAAAGATAACCTTAGGTGAAATCCGAGCGATGTACCCACGTAAACCACATCTGAATAAACCTGAGGTAACTCCTACAGATGACCAGCCTTCCGCTGAGACAATCGAAGGTGTCACTAAGCCTTCCCACTACATGCTGTTTGACGACATTGAGGCTATCGAAGTGATTGCTCGTTCAATGACCGTTGAGCAGTTCAAGGGATACTGCTTCGGTa      <  1:251164/298‑1 (MQ=255)`  
                                                                                                                                                                                                                                                                                                        `aataatGAAGGTCGCCCAGATTATTTGCATGGCTGCGGTATTCTCTGCTCCAATGTCCCTTGCCACCTCTGCCCCGCAAATAACGATCAAAAGATAACCTTAGGTGAAATCCGAGCGATGGACCCACGTAAACCACATCTGAATAAACCTGAGGTAACTCCTACAGATGACCAGCCTTCCGCTGAGACAATCGAAGGTGTCACTAAGCCTTCCCACTACATGCTGTTTGACGACATTGAGGCGATCGAAGTGATTGCTCGTTCAATGACCGTTGAGCAGTTCAAGGGATACTGCTTCGGTa      <  1:123184/301‑1 (MQ=255)`  
                                                                                                                                                                                                                                                                                                        `aataatGAAGGTCGCCCAGATGATTTGAATGGCTGCGGTATTCTCTGCTCCAATGTCCCTTGCCACCTCTGCCCCGCAAATAACGATCAAAAGATAACCTTAGGTGAAATCCGAGCGATGGACCCACGTAAACCACATCTGAATAAGCCTGAGGTAACTCCTACAGATGACCAGCCTTCCGCTGAGACAATCGAAGGTGTCACTAAGCCTTCCCACTACATGCTGTTTGACGACATTGAGGCTATCGAAGTGATTGCTCGTTCAATGACCGTTGAGCAGTTCAAGGGATACTGCTTCGGTa      >  1:51242/1‑301 (MQ=255)`  
                                                                                                                                                                                                                                                                                                          `taatGAAGGTCGCCCAGATGATTTGAATGGCTGCGGTATTCTCTGCTCCAATGTCCCTTGCCACCTCTGCCCCGCAAATAACGATCAAAAGATAACCTTAGGTGAAATCCGAGCGATGGACCCACGTAAACCACATCTGAATAAACCTGAGGTAACTCCTACAGATGACCAGCCTTCCGCTGAGACAATCGAAGGTGTCACTAAGCCTTCCCACTACATGCTGTTTGACGACATTGAGGCTATCGAAGTGATTGCTCGTTCAATGACCGTTGAGCAGTTCAAgggctgtctcttatacaca    >  1:388571/1‑285 (MQ=255)`  

                                                                                                                                                                                                                                                                                                            `|`                                                                                                                                                                                                                                                                                                              
`AGCGTCCACATCATTATCACCCGTGGTGACTTTGAGAAAGCAATAGACAAGAAACGTCCGGTTCTTAGTGTGGCAGTGACCCGCTTCCCGTTCGTCCGTCTGTTACTCAAACGAATCAAGGAGGTGTTCTGATGGGACTGTTAGATGGTGAAGCCTGGGAAAAAGAAAACCCGCCAGTACAAGCAACTGGGTGTATAGCTTGCTTAGAGAAAGATGACCGTTATCCACACACCTGTAACAAAGGAGCTAACGATATGACCGAACGTGAACAAGAGATGATCATTAAGTTGATAGACAATAATGAAGGTCGCCCAGATGATTTGAATGGCTGCGGTATTCTCTGCTCCAATGTCCCTTGCCACCTCTGCCCCGCAAATAACGATCAAAAGATAACCTTAGGTGAAATCCGAGCGATGGACCCACGTAAACCACATCTGAATAAACCTGAGGTAACTCCTACAGATGACCAGCCTTCCGCTGAGACAATCGAAGGTGTCACTAAGCCTTCCCACTACATGCTGTTTGACGACATTGAGGCTATCGAAGTGATTGCTCGTTCAATGACCGTTGAGCAGTTCAAGGGATACTGCTTCGGTAACAT  >  NC_001604.1/8035‑8635`

  

Alignment Legend

Aligned base mismatch/match (shaded by quality score): `ATCG/ATCG` `< 3 ≤` `ATCG/ATCG` `< 11 ≤` `ATCG/ATCG` `< 26 ≤` `ATCG/ATCG` `< 36 ≤` `ATCG/ATCG` `< 38 ≤` `ATCG/ATCG`

Unaligned base: `atcg`    Masked matching base: `atcg`    Alignment gap: `‑`    Deleted base: `‑`

Reads not counted as support for junction

read\_name Not counted due to insufficient overlap past the breakpoint.

read\_name Not counted due to not crossing MOB target site duplication.
